# Modeling Framework for Fracture in Multiscale Cement-Based Material Structures

**DOI:** 10.3390/ma10060587

**Published:** 2017-05-26

**Authors:** Zhiwei Qian, Erik Schlangen, Guang Ye, Klaas van Breugel

**Affiliations:** Faculty of Civil Engineering and Geosciences, Delft University of Technology, Delft 2628 CN, The Netherlands; erik.schlangen@tudelft.nl (E.S.); g.ye@tudelft.nl (G.Y.); k.vanbreugel@tudelft.nl (K.v.B.)

**Keywords:** parameter-passing upscaling, lattice fracture analysis, Anm material model, cement paste, mortar, concrete, irregular real-shape aggregates

## Abstract

Multiscale modeling for cement-based materials, such as concrete, is a relatively young subject, but there are already a number of different approaches to study different aspects of these classical materials. In this paper, the parameter-passing multiscale modeling scheme is established and applied to address the multiscale modeling problem for the integrated system of cement paste, mortar, and concrete. The block-by-block technique is employed to solve the length scale overlap challenge between the mortar level (0.1–10 mm) and the concrete level (1–40 mm). The microstructures of cement paste are simulated by the HYMOSTRUC3D model, and the material structures of mortar and concrete are simulated by the Anm material model. Afterwards the 3D lattice fracture model is used to evaluate their mechanical performance by simulating a uniaxial tensile test. The simulated output properties at a lower scale are passed to the next higher scale to serve as input local properties. A three-level multiscale lattice fracture analysis is demonstrated, including cement paste at the micrometer scale, mortar at the millimeter scale, and concrete at centimeter scale.

## 1. Introduction

Cement-based materials, such as cement paste, mortar, and concrete, are multiscale heterogeneous construction materials. Normal concrete is made from coarse aggregates (e.g., crushed stones, river gravels), fine aggregates (e.g., sands), cement, and water. The chemical reaction starts immediately when water is mixed with cement, and cement paste is produced. The cement paste keeps aggregates together, and forms a system which is able to carry loads. Mortar consists of cement paste and sands, and concrete is composed of mortar and coarse aggregates.

**Figure 1:**
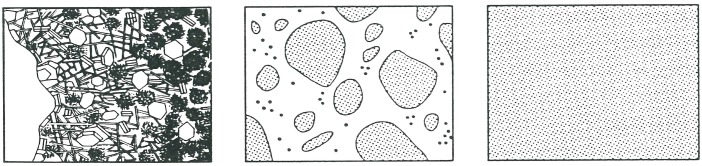
Three-level approach (micro, meso, and macro) according to Wittmann [1].

A three-level approach was proposed by Wittmann [1] for the study of multiscale phenomenon in cement-based materials, as shown in Figure 1. The three levels are: (a) the micro level, which concerns the microstructure of hardened cement paste; (b) the meso level, which deals with pores, inclusions, and cracks; (c) the macro level, which is related to the structural element, and concrete can be regarded as homogeneous materials at this level.

Cement-based materials have different fracture behaviors at different scales, due to different material structures at the level of interest. The mechanical performance of the material structure is determined by the distribution of the phases, and the local mechanical properties of an individual phase. They can be measured in the laboratory, as well as be simulated by computational models.

The concept of lattice was proposed by Hrennikoff in the 1940s to solve elasticity problems using the framework method [2]. In the 1970s, the lattice model was introduced to theoretical physics to study the fracture behaviors of disordered media. In the 1990s, Schlangen and van Mier started to adapt and apply this model to simulate the fracture processes in concrete [3]. Plenty of efforts were made to develop the lattice fracture model in a variety of different settings: regular/irregular network, triangular/quadrangular mesh, truss/beam element, the method to implement heterogeneity (random distribution of local properties/microstructure mapping), and whether to introduce the softening at the element level [4,5,6,7,8,9,10,11,12,13,14].

In the lattice fracture model, the continuum is replaced by a lattice network of beam elements. Subsequently, the microstructure of the material can be mapped onto these beam elements by assigning them different properties, depending on whether the beam element represents a grain or matrix. More details about the modeling procedures can be found in Section 3 in this paper. Various conventional laboratory experiments, such as uniaxial tensile tests, compressive tests, shear tests, bending tests, and torsional tests, can be simulated by the lattice fracture model, and the model can be applied towards a wide range of multiphase materials, such as cement paste, mortar, concrete, asphalt, and graphite.

The 3D lattice fracture model, which was implemented in [15,16,17], can simulate the fracture processes and crack propagation in cement-based materials, and thus, predict the global mechanical properties, such as Young’s modulus, tensile strength, and fracture energy.

**Figure 2:**
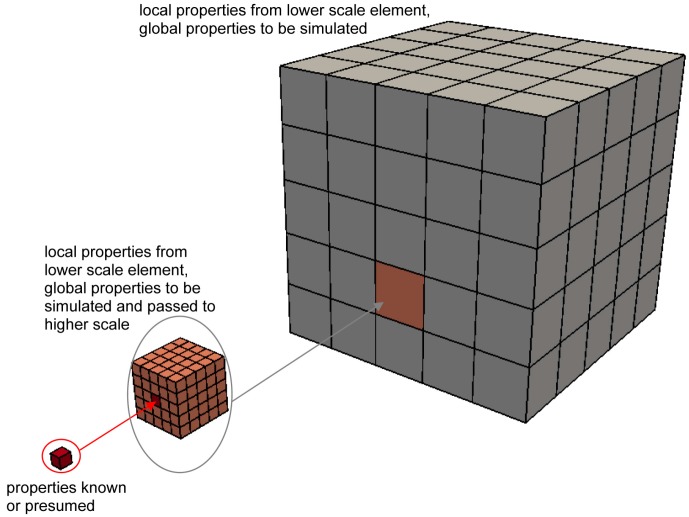
Parameter-passing multiscale modeling scheme.

A parameter-passing multiscale modeling scheme can be defined to link the fracture in cement paste at the microscale to the mechanical performance of concrete at the macroscale. The lattice fracture model is called three times to simulate the global mechanical properties of cement paste at the microscale, mortar at the mesoscale, and concrete at the macroscale, respectively. Each higher scale asks for local mechanical properties from its neighboring lower scale, and the global mechanical properties simulated at a lower scale are passed to the next higher scale to be used as inputs. Applying the parameter-passing scheme towards cement paste, mortar, and concrete yields the following modeling procedures. The mechanical properties of cement paste at the microscale, including its elastic properties and fracture properties, are simulated by the lattice fracture model, and then these properties are used as inputs to predict the mechanical properties of mortar at the mesoscale. After that, the simulated properties of mortar are passed to the macroscale to obtain the global mechanical performance of concrete. A schematic diagram of the parameter-passing multiscale modeling scheme is given in Figure 2.

## 2. Material Structures of Cement Paste, Mortar, and Concrete

The material structures of cement paste, mortar, and concrete are analyzed by the lattice fracture model at their respective scales, with part of the input parameters coming from lower-scale simulations. The methods to obtain these material structures are discussed in this section.

### 2.1. Microstructures of Cement Paste

The clinker of Portland cement is mainly composed of calcium, silicon, and oxygen. In cement chemistry, it is usually represented in terms of constituents as tricalcium silicate $3CaO\cdot SiO_{2}$ ($C_{3}S$), dicalcium silicate $2CaO\cdot SiO_{2}$ ($C_{2}S$), tricalcium aluminate $3CaO\cdot Al_{2}O_{3}$ ($C_{3}A$), and calcium ferroaluminate $4CaO\cdot Al_{2}O_{3}\cdot Fe_{2}O_{3}$ ($C_{4}AF$). A set of chemical reactions is initiated when water is mixed with cement, the process of which is called hydration. The hydration process is always accompanied by heat release, as the energy state of the cement mixture turns from a higher one to a lower one. The heat released indicates the degree of hydration, and it can be used as a measurement to determine the extent of hydration. The hydration products are also generated during the hydration process, which mainly include calcium silicate hydrates (CSH) and calcium hydroxides (CH).

The microstructure of cement paste can be obtained either experimentally or numerically. Micro X-ray Computed Tomography (micro CT) offers a non-destructive experimental technique to collect microstructure information of cement paste in terms of digitized voxels [18]. Computer modeling packages are also available to simulate the cement hydration and microstructure formation processes, for instance, the HYMOSTRUC3D model developed by TU Delft [19,20], the NIST CEMHYD3D toolkit [21], and the µic model by EPFL [22].

**Figure 3:**
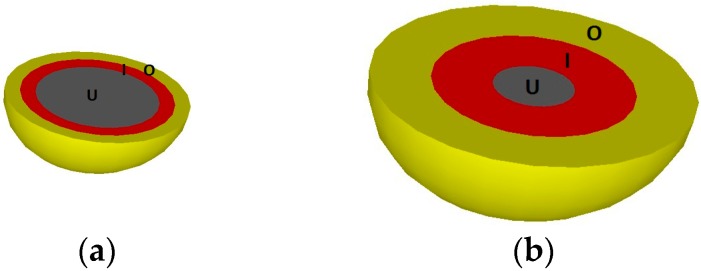
Hydration of a single cement particle (U: unhydrated cement, I: inner product, O: outer product): (**a**) earlier stage; and (**b**) later stage.

In the HYMOSTRUC3D model, the cement particles are modeled as spheres and these spherical particles grow during the hydration process. The inputs include the specimen size, the mineralogical composition of the cement, the cement fineness in terms of the Blaine value (Rosin-Rammler particle size distribution is assumed) and the water/cement ratio. The amount of hydration products is dependent on the degree of hydration. A simplification is made in the model that the amount of CH product is substituted with the same amount of the CSH product. In general, the hydrating cement particle contains three layers from the center to the outward surface, namely, unhydrated cement, inner product, and outer product, as shown in Figure 3a.

The cement grain dissolves and the hydration products are generated gradually during the hydration process, which yields expansion and layer thickness change of the cement particle, as shown in Figure 3b. The amount of unhydrated cement is decreasing, while the inner product and outer product are being produced. The interactions between the neighboring particles are taken into account in the HYMOSTRUC3D model. If the outer product of one hydrating cement particle meets the outer product of another particle, then the overlapping part is redistributed to the outer layer of the particles.

**Figure 4:**
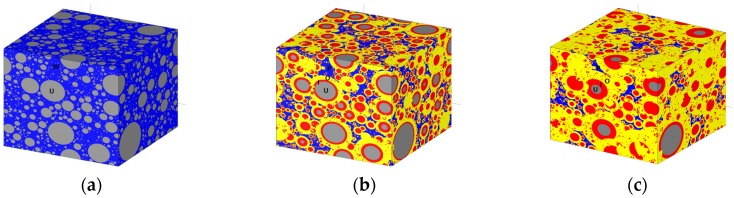
Microstructures of cement paste at different degrees of hydration simulated by the HYMOSTRUC3D model (U: unhydrated cement, I: inner product, O: outer product, W: water, P: pore): (**a**) initial state; (**b**) degree of hydration 69%; and (**c**) degree of hydration 88%.

The initial microstructure of cement paste can be created by parking multiple spherical particles into an empty container, as shown in Figure 4a, with a water/cement ratio equal to 0.4. The hydration reaction produces the inner and outer products. The microstructure of cement paste keeps changing during the hydration process, as shown in Figure 4b,c. The corresponding degrees of hydration are 69% and 88%, respectively. The size of the cement paste specimen is 100 µm, and periodic material boundary conditions apply.

### 2.2. Material Structures of Mortar and Concrete

Mortar can be made by putting sands into cement paste, and if some additional coarse aggregates are also inserted into the system, then it becomes concrete. From a modeling point of view, the material mesostructures of both mortar and concrete can be represented as particles embedded in a matrix. The particles are interpreted as sands, and the matrix as cement paste in the mortar model, while the particles are coarse aggregates, and the matrix is mortar in the concrete model. Therefore, it is achievable to make a universal material mesostructure model for mortar and concrete. However, the particle shape characterizations can be different for sands and coarse aggregates, furthermore, they can even be different for different classes of sands or coarse aggregates. This requires the universal material model to be able to recognize various particle shape characterizations. It is pointed out by Garboczi [23] that the spherical harmonics is an appropriate mathematical tool to characterize the shape of particles mathematically. The procedures to retrieve particle shape characterizations for a given class of aggregates based on CT (Computed Tomography) scanned digital images are also established in [23]. The next step is to place multiple irregularly-shaped particles into a pre-defined empty container to build up a complete model of particles embedded in a matrix, which is addressed in [15,24] and is named the Anm material model. The name of the model is derived from the symbol of spherical harmonic coefficients $a_{nm}$ The Anm material model is employed to simulate the material mesostructures of mortar and concrete, respectively, in this paper.

In the Anm material model, the concept of particles embedded in matrix applies. An empty container is created to represent a specimen at the beginning, and then all of the particles are placed one after one into this container, from the larger ones to the smaller ones. It is good to start with the largest particles as it would be more difficult to place them if they were processed at a later stage. All of the particles are separated into several sieve ranges, according to the particle sizes indicated by the particle widths. The largest sieve range is processed first. A width within this sieve range is picked up randomly, and then it is assigned to a particle which is chosen from the appropriate particle shape database. The particle shape database can be created for varying classes of powders and aggregates with the procedures proposed in [23]. An arbitrary rotation is performed on the particle to avoid possible orientation bias which might be introduced during the production of the particle shape database. After the rotation, the particle is placed at a randomly-chosen primary location in the specimen, and then the ghost locations are determined, if any, depending on the type of the specimen boundary conditions and the position of the particle. Those particles which reside at boundaries are mirrored to ghost locations in case periodic boundary conditions apply. See [15,24] for details about periodic material boundaries. The primary particle and its ghost particles are checked against all of the previously-placed particles for overlap. If no overlap is detected, then the particle enters the simulation box successfully, otherwise it will be moved to a new randomly-chosen location. The reassignment of the location is subject to a pre-defined maximum number of attempts. After the consecutive failures reach the limit, the particle will be resized to another randomly-selected width within the current sieve range, and then be thrown into the specimen following the same trial-and-error procedure. The particle size rescale is also subject to a pre-defined maximum number of attempts. If the rescales do not help, then the particle will be rotated to have another orientation. If the problem still exits, then a new shape will be chosen from the particle shape database. In the case that the particle cannot find its position eventually, it may suggest that there is no space available for new particles within the current sieve range any more. The next sieve range will be processed when no availability for the current sieve range is found, or when all of the particles within the current sieve range have already been placed. The above trial-and-error procedures are called parking procedures. Some other parking/packing models are also available in the literature that might lead to denser material structures, such as the dynamic particle packing by Stroeven [25]. The Anm model was later improved by Thomas et al. [26].

**Figure 5:**
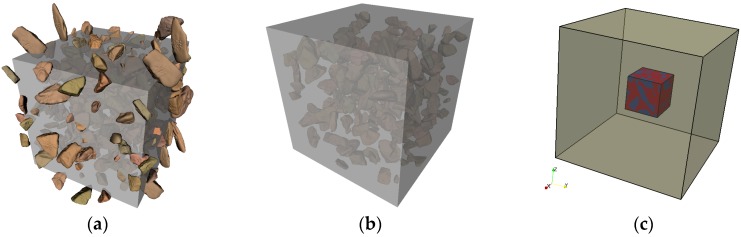
Material mesostructures of mortar and concrete simulated by the Anm material model: (**a**) 10 mm mortar specimen with periodic material boundaries; (**b**) 150 mm concrete specimen with mold material boundaries; and (**c**) 40 mm cutting-out of the 150 mm concrete specimen.

The mesostructure of mortar is simulated for a cubic specimen of the size 10 mm, and it is represented by sand particles embedded in a cement paste matrix. A periodic material boundary condition is employed, which mirrors the sticking-out particles to the opposite surfaces, as this mortar represents a randomly-chosen material phase inside concrete. The Anm material model parks all of the sand particles into the empty simulation box with periodic material boundaries one after one, ensuring that there is no overlap between any of the two particles. The resulting mesostructure of the 10 mm cubic mortar specimen with irregularly-shaped sand particles is sketched in Figure 5a.

A concrete specimen of the size 150 mm in cubic shape is simulated, as shown in Figure 5b. The specimen has two phases, the mortar matrix and the irregularly-shaped crushed stone aggregates. The mold material boundary applies, which requires all of the particles to be inside the specimen and no part of a particle can pass through a surface.

A smaller piece of concrete 40 mm in size is cut out from the original simulated 150 mm concrete specimen at its center, as shown in Figure 5c, in order to reduce the computational cost for the subsequent lattice fracture analysis. However, the cut would introduce some scatter in the simulated mechanical performance of concrete, as a smaller specimen contains less aggregates; moreover, the size of the specimen and the size of the largest aggregate it contains are getting closer as well.

## 3. Mechanical Performance Evaluation of Cement Paste at the Microscale

The 3D lattice fracture model is employed to evaluate the mechanical performance of cement paste at the microscale, based on the microstructures of cement paste which are provided by the HYMOSTRUC3D model. The microstructures of cement paste, in terms of spherical particles, are converted into voxel-based digital images. After that, a lattice network is constructed on the basis of the digital image, which represents the microstructure of cement paste. The lattice construction procedures are discussed in [15,16].

The specimen of the size 100 µm is meshed at the resolution 1 µm/voxel, and a 3D hexahedral lattice network is constructed. A cell is created within each voxel sharing the same center, and the length ratio of the cell to the voxel is defined as randomness. The value of randomness is always between 0 and 1. More details about the randomness can be found in [15]. A lattice node is chosen within the cell randomly. Neighbor nodes are connected by a lattice element. In the lattice mesh of cement paste at the microscale, the randomness of the lattice system is set to 0 for all of the boundary cells and 0.5 for other cells. This configuration would yield a realistic crack pattern and a regular specimen shape. The cross-section of the lattice element is assumed to be circular, and its area is equal to the perpendicular voxel surface area which is 1 µm^2^ in this simulation example.

The elastic properties of solid phases can be measured or simulated as presented in [27,28], the values of which are scattered due to different measurement approaches used. The tensile strength ratio of each phase 1.8:0.24:0.15 is equal to the measured nanoindentation hardness ratio 12:1.6:1. The measured and assumed local mechanical properties of individual solid phases are given in Table 1.

The assignment of local mechanical properties to a lattice element is related to the type of the lattice element in question, which is determined by the locations of its two nodes. If both ends of an element are located in the same phase, then this element is assigned the same mechanical properties as the phase in question, otherwise it is classified as an interface element. The mechanical properties of an interface element are preferred to be measured in laboratory test, but if it lacks experimental data, the following guidelines may be applied. The Young’s modulus of an interface element $E_{I}$ is the harmonic average of the Young’s moduli of the grain $E_{G}$ and the matrix $E_{M}$, which reads $E_{I}=\frac{2E_{G}E_{M}}{E_{G}+E_{M}}$. The tensile strength of an interface element takes the lower value of the two phases of the grain and the matrix, because failure takes place at the weakest location. Shear modulus of an interface element is determined in a similar way to the determination of its Young’s modulus. Three solid phases in the microstructure result in six types of lattice elements, as listed in Table 2. No lattice node is generated in the voxels which represent pore phase, as it does not contribute to the global mechanical performance of the specimen. All lattice elements behave as linear-brittle, and the Young’s modulus, shear modulus, and tensile strength are given in Table 3.

**Figure 6:**
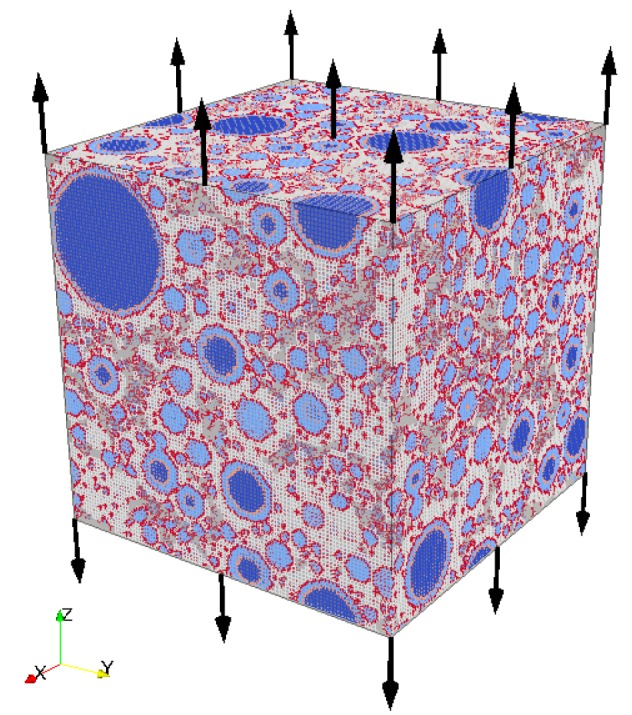
Simulated uniaxial tensile test on the lattice system of cement paste at the microscale.

A conventional uniaxial tensile test is simulated on the lattice system. The external tensile load is imposed on the top and bottom surfaces in the z-direction, and all the other surfaces are free to expand and/or shrink, as shown in Figure 6.

**Figure 7:**
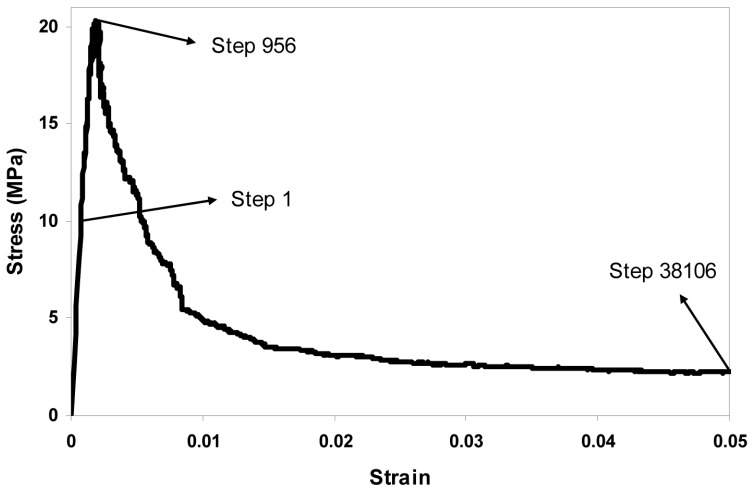
Simulated tensile stress-strain diagram of cement paste at the microscale.

**Figure 8:**
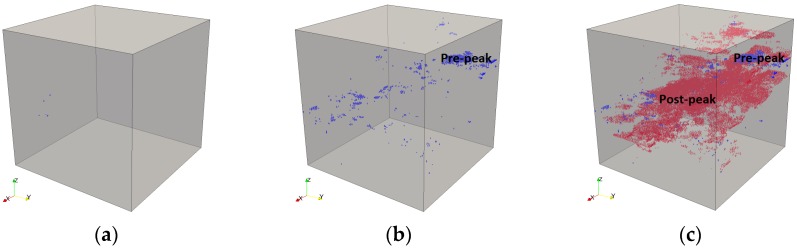
3D microcracks propagation of cement paste due to tension at the microscale, corresponding with Figure 7: (**a**) microcracks initiation (step 5); (**b**) microcracks at peak load (step 956); and (**c**) microcracks in the final failure state (step 38,106).

The fracture process is simulated by the removal of lattice elements step by step. The basic idea of lattice fracture analysis is that of imposing a prescribed displacement on the lattice structure, finding the critical element that has the highest stress/strength ratio, and then removing it from the system. At every analysis step, a unit displacement is first imposed, and the reaction force is calculated. Then the displacement and the force are scaled linearly to make the weakest element just broken. The corresponding pair of displacement and force is recorded and becomes a point on the final simulated load-displacement curve for the system. This procedure is repeated until the system fails, and these multiple steps produce multiple points. These points are connected to form the load-displacement diagram, which can be converted to a stress-strain diagram, as shown in Figure 7. The sequentially-removed lattice elements indicate the microcrack propagation process, as shown in Figure 8.

The stress-strain diagram reveals the tensile behavior of cement paste at the microscale, from which the elastic modulus, tensile strength, strain at peak load, and fracture energy can be obtained. The Young’s modulus is the slope of the curve at the linear stage in the stress-strain diagram in Figure 7, the tensile strength corresponds to the peak point, and the fracture energy can be computed as the length in the loading direction multiplied by the area below the stress-strain curve. The absolute values of the global tensile strength and the strain at peak load are linearly dependent on the local input strength, so if all of the local input strengths would be doubled, then the resulting global tensile strength and the strain at peak load would also be doubled, and the fracture energy would be four times larger as it is related to the square of the local strength values.

In Figure 8, it shows that first a few microcracks are initiated around the middle part of the specimen, and then the microcracks spread further until the final failure state. In total 38,106 lattice analysis steps are performed, which means 38,106 lattice elements are broken, and thus, are removed from the system, as all elements behave as linear-brittle locally. The pre-peak and post-peak microcracks are also shown in Figure 8.

## 4. Upscaling from Cement Paste to Mortar, and then to Concrete

The material structures of cement paste are simulated by the HYMOSTRUC3D model, and mortar and concrete are simulated by the Anm material model, respectively, in Section 2. The mechanical properties of cement paste are predicted by the 3D lattice fracture model in Section 3. In this section the simulated properties of cement paste are passed to the mesoscale for the mechanical performance evaluation of mortar, and then the upscaling is performed again from mortar to concrete, using the 3D lattice fracture model with the parameter-passing scheme.

The scale division of cement paste, mortar and concrete is given in Table 4. Some conditions need to be satisfied when determining the scale division. The specimen size should be at least 2.5 times larger than the largest particle, and the mesh size should be smaller than the smallest particle. The connecting length between cement paste and mortar is 100 µm, thus, the upscaling can be performed seamlessly, as demonstrated in Section 4.1. However, there is some length scale overlap between mortar and concrete, and the block-by-block technique is employed to solve this problem, which will be elaborated below.

### 4.1. Connecting Cement Paste to Mortar

**Figure 9:**
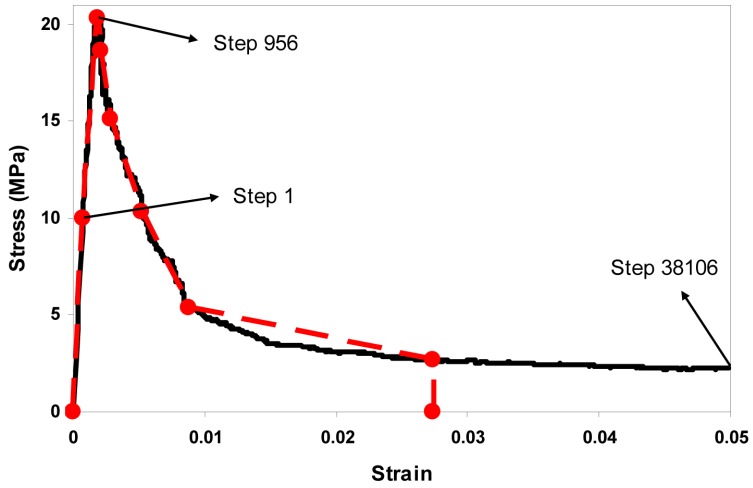
Approximation of non-linear stress-strain response by piece-wise linear curve (the black solid line represents the original simulated stress-strain curve, and the red dashed line represents the piece-wise linear approximation).

The microstructure of cement paste of the size 100 µm is simulated by the HYMOSTRUC3D model, as shown in Section 2.1, and then its tensile mechanical performance is evaluated by the 3D lattice fracture analysis, as demonstrated in Section 3. The resulting stress-strain curve is approximated by a piece-wise linear curve, as shown in Figure 9. The piece-wise linear curve serves as the input local mechanical properties of cement paste for the mortar properties prediction at the mesoscale. The points should be chosen in such a way that it makes the change of input properties gradual in terms of Young’s modulus and tensile strength, as listed in Table 5.

**Figure 10:**
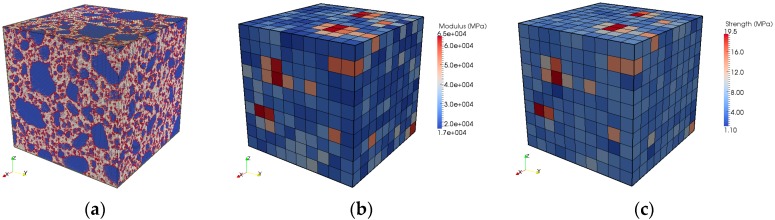
Mortar specimen of the size 10 mm at the mesoscale: (**a**) the lattice mesh at a size of 0.1 mm; (**b**) the simulated Young’s modulus of every block 1 mm in size; and (**c**) the simulated tensile strength of every block of the size 1 mm.

The mesostructure of mortar of the size 10 mm is simulated by the Anm material model, as shown in Section 2.2. The resulting mesostructure in Figure 5a is then digitized to facilitate the subsequent lattice network construction. In the mesostructure of mortar, two solid phases are present, namely cement paste and sand. The lattice mesh size is 0.1 mm, as shown in Figure 10a, ensuring that the properties of the cement paste can be passed to the mesoscale modeling seamlessly from the microscale modeling. Three types of lattice elements are defined during the lattice network mesh, which represent sand, cement paste, and interface elements, respectively. The local mechanical properties of sand and interface elements can be measured in the laboratory. The properties of cement paste are given in Figure 9, which are resulted from the microscale modeling. Table 6 summarizes the local mechanical properties of sand, cement paste, and interface elements in mortar at the mesoscale.

Rather than directly evaluating the global mechanical performance of the 10 mm mortar, the lattice system in Figure 10a is decomposed to a network of blocks. The size of a single block is 1 mm, thus, there are 10 blocks per direction in the original lattice system and in total 1000 blocks. This is done to provide inputs for the concrete level simulation, because the concrete specimen is meshed at 1 mm. Uniaxial tensile tests are simulated on these blocks one after one, using the local mechanical properties in Table 6 [3]. The resulting mechanical responses are scattered as the material structures of blocks may differ significantly. The simulated Young’s modulus and tensile strength of every block are shown in Figure 10b,c.

The simulated Young’s modulus of a 1 mm block is in the range of 17–65 GPa and the average value over these 1000 blocks is 29 GPa. The tensile strength is in the range of 1.1–19.5 MPa and the average value is 5.8 MPa. The stress-strain responses of these blocks are randomly passed onto the concrete mesomechanical modeling and serve as inputs there, as elaborated in Section 4.2.

### 4.2. Upscaling from Mortar to Concrete

The block-by-block technique is employed for the upscaling from mortar to concrete, due to the length scale overlap between them, as indicated in Table 4. The mortar specimen of 10 mm is decomposed into a network of 1 mm blocks, and then these blocks are evaluated by the 3D lattice fracture model one after one to get stress-strain responses, as demonstrated in Section 4.1.

**Figure 11:**
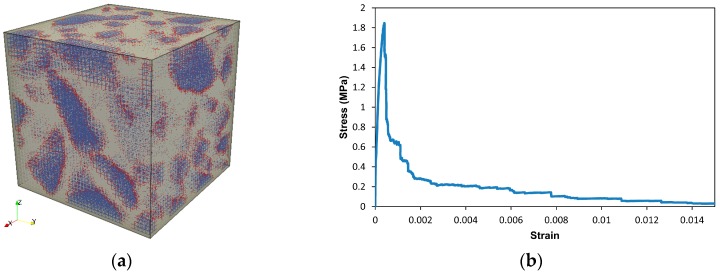
Concrete specimen of the size 40 mm at the mesoscale: (**a**) lattice mesh at a size of 1 mm; and (**b**) the simulated stress-strain response due to uniaxial tensile test on the lattice system.

Having obtained the mechanical properties of the 1 mm mortar blocks, the concrete mesomechanical modeling can now proceed. The mesostructure of the concrete is simulated by the Anm material model, as presented in Section 2.2. The 40 mm concrete specimen is then digitized at a resolution of 1 mm, and consists of two solid phases, namely stone and mortar. A lattice network is constructed based on the digital concrete specimen, and three types of lattice elements are identified, which represent crushed stone, mortar, and interface elements, respectively, as shown in Figure 11a. The local mechanical properties are given in Table 7 [3]. The properties of the lattice elements representing mortar are randomly picked up out of those 1000 mortar blocks to reflect the fact that the property of mortar is not a constant value, but in a range of varying values.

A uniaxial tensile test is simulated on the lattice system meshed from the 40 mm concrete specimen as shown in Figure 11a, using the local mechanical properties listed in Table 7. The resulting stress-strain response is presented in Figure 11b, and some mechanical properties can be computed as given in Table 8.

The pattern of the simulated stress-strain response of the concrete specimen of the size 40 mm is similar to the one observed in laboratory, and the mechanical properties computed from the stress-strain diagram are also located within the reasonable range [29].

## 5. Summary, Discussion and Outlook

Cement-based materials, such as cement paste, mortar, and concrete, are multiscale heterogeneous construction materials. In this paper, the parameter-passing multiscale modeling scheme is established and applied to simulate fracture mechanisms.

The starting point of the fracture simulations is the microstructure of hardened cement paste at the microscale. It is assumed that the local mechanical properties are brittle at this scale. This is an arbitrary assumption. The properties at this scale are difficult to measure experimentally, and therefore, there is no proof yet for this assumption of brittle behavior at the microscale. Perhaps it is necessary to go to the nanoscale, or even to the atomic scale, to have real local brittle behavior, but at least it seems reasonable to assume that the behavior becomes more brittle at lower scales.

At the microscale, the local mechanical properties can be determined with the help of a nanoindentation test. The test gives local stiffness and hardness, which can be related to the tensile strength of the components. In this paper, the absolute values of the tensile strength are not measured directly by the test, but the tensile strength ratios of the components are used to assume the tensile strength values of the components at the microscale. The real tensile strength values can be corrected after simulations on a level (mesoscale or macroscale) where mechanical tests can be performed easily. At the microscale, some attempts have been made to conduct direct mechanical tests on the composites, and they seem quite successful [30].

In the multiscale modeling scheme presented in this paper, the block-by-block technique, is employed to solve the length scale overlap between mortar and concrete. The material structures of cement paste are simulated by the HYMOSTRUC3D model, and mortar and concrete are simulated by the Anm material model, respectively. The 3D lattice fracture model is used to evaluate their mechanical performance by simulating a uniaxial tensile test. The simulated output properties at a lower scale are passed to a higher scale to be served as input local properties, thus, a three-level multiscale lattice fracture analysis is demonstrated.

Bažant concluded that the only valid approach was a discrete (lattice-particle) simulation of the mesostructure of the entire structural region, in which softening damage could occur [31]. However, this problem was later overcome by Nguyen et al. [32]. In this paper, the material structures of cement paste, mortar, and concrete are based on particles embedded in matrix model. The localization limiter of fracture energy is enabled by the inclusion of particles in the material structures. Furthermore, the simulated softening behavior of cement paste at micrometer scale is transferred to millimeter scale for mortar level simulation. The obtained softening behavior of mortar at the millimeter scale is passed to the centimeter scale for concrete-level simulation. This implies that the softening behavior of a material at a lower scale should be taken into account in a higher-scale simulation.

The proposed model will be adopted in the future to simulate fracture at multiple scales in different geometries. For example, the sample size, aggregate shapes, and boundary conditions are taken the same as in the laboratory. An experimental campaign has started to perform tests on different levels and different materials. This would be able to validate the modeling procedure further, and to obtain proof for the assumed local material properties.

## Figures and Tables

**Table 1:** Measured and assumed local mechanical properties of solid phases of cement paste [27,28].

No.	Solid Phase	Young’s Modulus E (GPa)	Shear Modulus G (GPa)	Tensile Strength f_t_ (GPa)
1	Unhydrated cement	135	52	1.8
2	Inner product	30	12	0.24
3	Outer product	22	8.9	0.15

**Table 2:** Classification of lattice element types in cement paste.

No.	Element Type	Node 1 Phase	Node 2 Phase
1	Unhydrated cement	Unhydrated cement	Unhydrated cement
2	Inner product	Inner product	Inner product
3	Outer product	Outer product	Outer product
4	Interface U-I	Unhydrated cement	Inner product
5	Interface I-O	Inner product	Outer product
6	Interface O-U	Outer product	Unhydrated cement

**Table 3:** Derived local mechanical properties of lattice elements in cement paste.

No.	Element Type	Young’s Modulus E (GPa)	Shear Modulus G (GPa)	Tensile Strength f_t_ (GPa)
1	Unhydrated cement	135	52	1.8
2	Inner product	30	12	0.24
3	Outer product	22	8.9	0.15
4	Interface U-I	49	20	0.24
5	Interface I-O	25	10	0.15
6	Interface O-U	38	15	0.15

**Table 4:** Scale division and specifications of the cement paste, mortar and concrete specimens.

	Cement Paste	Mortar	Concrete
Specimen size	100 µm	10 mm	40 mm
Lattice mesh size	1 µm	0.1 mm	1 mm
Minimum particle size	1 µm	0.125 mm	4 mm
Maximum particle size	37 µm	4 mm	16 mm

**Table 5:** Mechanical properties of the 100 µm cement paste, corresponding with Figure 9.

Point	P1	P2	P3	P4	P5	P6	P7
Young’s modulus E (MPa)	12,846	11,096	7601	3627	1590	611	87
Shear modulus G (MPa)	5265	4548	3115	1486	652	250	36
Tensile strength f_t_ (MPa)	10	20	18.6	15.1	10.3	5.4	2.7

**Table 6:** Measured and derived local mechanical properties of lattice elements in mortar [3].

No.	Element Type	Young’s Modulus E (GPa)	Shear Modulus G (GPa)	Tensile Strength f_t_ (MPa)
1	Sand (uncrushed)	70	29	24
2	Cement paste	Piece-wise linear, see Table 5		
3	Interface	22	8.9	0.75

**Table 7:** Measured and derived local mechanical properties of lattice elements in concrete [3].

No.	Element Type	Young’s Modulus E (GPa)	Shear Modulus G (GPa)	Tensile Strength f_t_ (MPa)
1	Stone (crushed)	70	29	24
2	Mortar	Piece-wise linear and varied based on the 1 mm mortar blocks		
3	Interface	41	17	1

**Table 8:** Simulated mechanical properties of concrete at mesoscale, corresponding with Figure 11b.

Young’s Modulus E (GPa)	Tensile Strength f_t_ (MPa)	Strain at Peak Load ε_p_	Fracture Energy G_F_ (N/mm)
31	1.8	0.04%	0.127
